# The Facilitative Effects of Creative Cognitive Reappraisal on Teachers' Emotion Regulation

**DOI:** 10.1002/pchj.70030

**Published:** 2025-07-15

**Authors:** Li Chen, Yuan Yao, Bin Wang, Yanming Hou, Jing Luo, Xiaofei Wu

**Affiliations:** ^1^ Zhejiang Philosophy and Social Science Laboratory for Research in Early Development and Childcare Hangzhou Normal University Hangzhou China; ^2^ Department of Psychology Capital Normal University Beijing China; ^3^ Department of Psychology Hangzhou Normal University Hangzhou China

**Keywords:** creative cognitive reappraisal, emotion regulation, IAPS, negative emotion, pleasure, teacher, TNESS

## Abstract

Creative cognitive reappraisal is an emerging emotion regulation strategy, but existing experimental studies often lack ecological validity due to two key limitations: the challenge of spontaneously generating creative cognitive reappraisal and the passive presentation of materials, which resembles comprehension rather than active application. This study addresses these gaps by investigating the teachability and effectiveness of creative cognitive reappraisal in real‐world contexts. Using a 3 × 2 mixed‐factorial design, 82 teachers provided two personal negative events at baseline and were randomly assigned to one of three conditions (creative cognitive reappraisal, ordinary cognitive reappraisal, and positive emotional picture). Participants were trained in their assigned emotion regulation strategy based on a learning‐test paradigm, using materials from the International Affective Picture System and Teachers' Negative Emotional Scenarios System. Pleasure was measured at two time points: immediately after the learning phase and 3 days later, using 20 common and two personal negative teacher‐related scenarios. Qualitative data on insights gained from the learning phase were also collected. For common negative events, creative cognitive reappraisal demonstrated a meaningful, delayed, and significant effect after 3 days. The creative cognitive reappraisal group also generated the most creative reappraisal interpretations, highlighting its unique efficacy. These findings suggest that creative cognitive reappraisal is a teachable and enduring skill with delayed benefit for regulating negative emotions in real‐world contexts. It highlighted the importance of allowing time for emotional processing—rather than attempting immediate regulation—which could create a pathway for more effective regulation later.

## 1 Introduction

Teachers play multifaceted roles and face unique emotional and occupational stressors, including high work pressure, low psychological satisfaction, and the challenges of role transitions, which can threaten their mental health and teaching performance (Chang 2009; Frenzel et al. 2009). Teaching involves not only the transmission of knowledge and skills, but also the management of emotions, as teachers frequently experience a wide range of emotions while interacting with students, colleagues, administrators, and parents (Wang et al. 2023). Research shows that negative emotions, such as anger and frustration, can significantly impair teachers' well‐being, motivation, and instructional behaviors (Burić and Frenzel 2019; Frenzel et al. 2009). Given these high emotional demands, effective emotion regulation is crucial for supporting teacher well‐being, sustaining motivation, and enhancing classroom management.

Emotion regulation refers to an individual's attempt to choose when and what emotions to have and how to experience or express those emotions (Gross 2015). Cognitive reappraisal, as an effective emotion regulation strategy, involves changing individual emotions by constructing one's perception of the current situation by changing one's mental set and original information processing mode of negative stimuli (Webb et al. 2012; Ochsner and Gross 2005). Building upon the concept of cognitive reappraisal, Wu et al. (2017) have introduced an efficient emotion regulation strategy known as creative cognitive reappraisal (CCR).

CCR is defined as a strategy that employs innovative and insightful reinterpretations of negative stimuli or situations, offering novel and appropriate perspectives to reshape the understanding of these events. For example, when looking at an image of a filthy toilet bowl with some leftover food or waste inside, CCR might interpret it as “*A woman, who has been longing to become pregnant, just found out she is expecting a baby, and she is overwhelmed with joy*” (Wu et al. 2019). The mess in the toilet could be reinterpreted as a result of morning sickness, a common symptom of pregnancy, transforming the unpleasant image into a symbol of her happiness and new beginnings. The development of CCR strategies has shown promising results in inducing more comprehensive changes in representations and enhancing the effectiveness of emotion regulation.

The lab behavioral results showed that (1) in terms of immediate regulatory effects, creative reappraisal led to more positive evaluations of standardized negative images, functioning as a strategy that shifts emotions from negative to positive; (2) creative reappraisal also had a sustained effect in reducing negative emotions, with strong effects observed even after 3 days. This research underscores the pivotal role of creative insight in reappraisal and presents a novel, highly efficient emotion regulation strategy (Wu et al. 2019). However, validating the efficacy of CCR for emotion regulation presents a significant challenge. Emotions are inherently context‐dependent, shaped by the situation and individuals involved (Lazarus 2006); the effectiveness of CCR in real‐world contexts is still unclear.

Previous research (Wu et al. 2017, 2019) provided participants with pre‐constructed cognitive reappraisal sentences, which reflects the “comprehending” phase of CCR rather than genuine emotional regulation (Wu et al. 2022). Besides, the content of the materials was far away from the teachers' daily life. Moreover, generating creative reappraisal independently is difficult: only 31.9% of self‐generated reappraisals were rated as highly creative by the participants, and just 0.5% received similar ratings from expertise. This creates a dilemma: while individuals struggle to generate creative reappraisal on their own, pre‐constructed materials may not fully engage the emotional regulation process, limiting their real‐world application.

To address the limitation of previous studies and enhance ecological validity, this study introduces improvements to test the efficacy of creative reappraisal on emotion regulation. Recognizing the importance of emotion regulation for teachers, we developed the Teachers' Negative Emotional Scenarios System (TNESS; Chen et al. 2022), a database of 70 realistic negative scenarios commonly experienced by primary and secondary school teachers. These scenarios encompass challenges related to teacher‐student relationships, colleague interactions, work‐related stress, family concerns, and personal development. By utilizing the TNESS, this study provided authentic negative emotional contexts tailored to teachers, enabling a more valid assessment of creative reappraisal in reality.

We employed a learning‐test experimental paradigm to help participants adopt creative reappraisal within a short intervention and rigorously examine its impact on teachers' emotion regulation. This approach solves the difficulty individuals face in generating creative reappraisal on their own, as well as the limitations of passively comprehension prepared material without actively applying it.

Drawing on the concept of learning effect transfer (Singley and Anderson 1988), which refers to applying knowledge from one task to another, we aimed to demonstrate that the effectiveness of CCR stems from both creativity and cognitive reappraisal. To isolate the effect of creativity, we compared the CCR group with the ordinary cognitive reappraisal (OCR) group. To examine the unique contribution of cognitive reappraisal, we compared the CCR group with the positive emotional pictures (PEP) group. This design allows us to disentangle the roles of creativity and reappraisal in enhancing emotion regulation.

Our experiment consisted of two phases: the learning phase and the testing phase. Beforehand, participants completed self‐report baseline measures and recorded two personal negative events. Then they were randomly assigned to one of three groups: CCR, OCR, or positive emotional pictures (PEP). In the learning phase, participants learned different reappraisal strategies. In the testing phase, participants regulated emotions and rated their pleasure in response to common and personal negative events, with follow‐up testing 3 days later to assess long‐term effects.

We hypothesized that (H1) the CCR is more powerful than the OCR and positive emotional pictures in enhancing the pleasure; (H2) the similar effect exists in the long term.

## 2 Methods

### 2.1 Design

This study conducted a 3 (between participants: CCR/OCR/positive emotional picture) × 2 (within participants: immediately/3 days later) mixed‐factorial design. The independent variables were condition and time, the dependent variables are pleasure ratings both on teachers' common and personal negative scenarios.

### 2.2 Participants

The target sample size was 72, calculated using G*Power (*α* = 0.05, power (1 − *β*) = 0.85), with a large effect size (*f* = 0.4) according to Cohen (1988), based on a 3 × 2 mixed‐factorial design. A total of 117 teachers were recruited from schools in east‐central China, they were randomly assigned to one of three groups, with 39 individuals in each group. The exclusion criteria included: (1) incomplete participation in the experiment, (2) baseline anxiety or depression scores exceeding 71 or 0.7 respectively. After exclusion, the final sample included 82 participants: CCR (*n* = 27), OCR (*n* = 26), or positive emotional picture condition (*n* = 29). This experiment was approved by the Ethics Committee of Hangzhou Normal University.

### 2.3 Materials

The experimental materials consisted of three components: learning materials, testing materials, and measures. The learning materials were selected from the International Affective Pictures System (IAPS) and paired with respective reappraisal instructions. The testing materials were drawn from the TNESS. Measures included baseline self‐report mood, the self‐rating anxiety scale (SAS), the self‐rating depression scale (SDS) and two rounds of pleasure rating.

#### 2.3.1 Learning Materials

Learning materials were selected from the IAPS (Lang et al. 2008). For the creative and ordinary reappraisal group, 22 negative pictures were paired with distinct levels of creative reappraisals (Wu et al. 2019). For the positive emotional groups, 22 positive pictures were used.

##### 2.3.1.1 CCR Group

The 22 negative pictures depicted a variety of stimuli, (e.g., scary animals, disgusting objects, threatening accidents), and were paired with high‐level creative reappraisals (Wu et al. 2019). Mean rating for the 22 pictures on a 9‐point scale were: valence = 2.56 (SD = 0.52; range: 1.51–3.55), and the arousal = 5.43 (SD = 0.85; range: 3.93–7.35).

##### 2.3.1.2 OCR Group

**FIGURE 1:**
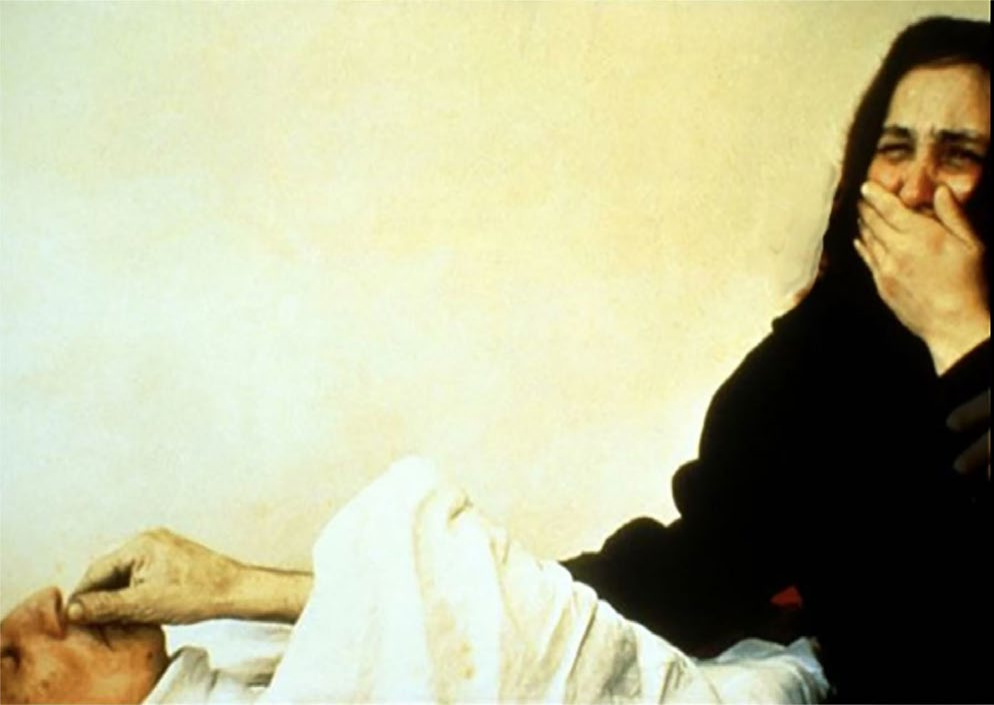
An example of reappraisal descriptions.

The same 22 negative pictures were paired with low‐level creative reappraisal group. This group served to control the reappraisal and validate the powerful role of creativity in CCR. Both two reappraisal instructions were matched in length and structure. For examples (see Figure 1):

Creative cognitive reappraisal: *“Older man: Did you fart? Daughter: No, I did not. Older man: Who did? Daughter: Your feet.”*

Ordinary cognitive reappraisal: *“The older man, finally woke up from a coma after the operation. His daughter, teared with happiness and gratitude.”*

##### 2.3.1.3 Positive Emotional Picture Group

Twenty‐two positive IAPS pictures (depicting warm human relationships, cute animals, and beautiful scenery) were used. Mean rating for the 22 pictures on a 9‐point scale were as follows: valence = 7.16 (SD = 0.32; range: 6.00–8.90), and the arousal = 5.56 (SD = 0.42; range: 3.62–7.64). These pictures served to control the positive emotions and validate the unique role of cognitive restructuring in CCR.

#### 2.3.2 Testing Materials

Testing materials were drawn from the TNESS (Chen et al. 2022). Forty scenarios were divided into two parallel sets: 20 for T1 (immediately after learning phase) and 20 for T2 (3 days later). Scenarios were balanced across emotional sources (e.g., students, parents, colleagues, family, work and self) and dimensions (valence, arousal, dominance and frequency). Parallel scenes were used to avoid response bias from repeated exposure.

Mean ratings for the 40 scenes on a 9‐point scale were: valence = 3.41 (SD = 0.26; range: 2.99–3.97), arousal = 5.20 (SD = 0.25; range: 4.53–5.66), dominance = 5.30 (SD = 0.24; range: 4.91–6.04) and frequency = 3.91 (SD = 0.60; range: 2.64–5.10). One scenario as an example is “*The final exam is approaching, and you are busy reviewing with your students. The boss asks you to mark another class's paper because a teacher is absent*.”

#### 2.3.3 Measures

##### 2.3.3.1 Baseline Measures

###### 2.3.3.1.1 Self‐Report Mood Scale

Participants rated seven specific moods (joy, calm, interest, anxiety, worry, anger, and disappointed) on a 5‐point scale from 1 (*not at all*) to 5 (*very much*) (Keller et al. 2014).

###### 2.3.3.1.2 Self‐Rating Anxiety Scale (SAS; Zung 1971)

This 20‐item scale assessed anxiety level over the past week, with each item scored on a 4‐point scale from 1 (*not at all*) to 4 (*very much*). Higher scores indicated greater anxiety, with scores below 50 considered normal and scores above 70 indicating severe anxiety. The scale demonstrated good internal consistency (*Cronbach's α* = 0.88).

###### 2.3.3.1.3 Self‐Rating Depression Scale (SDS; Zung 1965)

This 20‐items scale assessed depression level over the past week, with each item scored on a 4‐point scale from 1 (*not at all*) to 4 (*very much*). Higher scores indicated greater depression, with scores below 0.5 considered normal, and scores above 0.7 indicating severe depression. The scale showed excellent internal consistency (*Cronbach's α* = 0.90).

##### 2.3.3.2 Dependent Measures

###### 2.3.3.2.1 Self‐Report Pleasure

Pleasure was measured at each time point using a Visual Analog Scale (VAS). Participants rated their pleasure after regulating their emotions toward negative events on a scale from 0 (*not at all*) to 9 (*very much*). The average scores from 20 common negative scenes and two personal negative events were used to derive common scenes pleasure and personal scenes pleasure, respectively.

###### 2.3.3.2.2 Structured Questionnaire

Three open‐ended questions were as follows: (a) How did you regulate your emotions during the emotion regulation phase? (b) Did the pictures or interpretations from the learning phase influence your emotion regulation? (c) Did you gain any insights from the learning phase? If so, what are they? This approach allowed us to capture the qualitative richness of the insights gained from each intervention group.

### 2.4 Procedure

Participants were randomly assigned to one of three groups: CCR, OCR and positive emotion picture (PEP). They provided informed consent and completed baseline self‐report measures. Then, they wrote down two negative events that had affected their mood within the past month, with respective pleasure. The experiment consisted of a learning phase and a testing phase.

#### 2.4.1 Learning Phase

In the CCR and OCR groups: participants viewed 22 negative pictures, each paired with either a creative or ordinary reappraisal interpretation. Each trial began with a fixation cross (“+”) presented at the center of the screen for 1 s, followed by a negative picture displayed for 2 s. The corresponding reappraisal interpretation then appeared for 12 s, during which participants were instructed to comprehend the picture based on the interpretation. Afterwards, participants rated the creativity and effectiveness of the interpretation within 10 s. Once all 22 trials were completed, a 5‐min rest period followed. To control for order effects, half of the participants rated creativity first, while the other half rated effectiveness first.

In the PEP group, participants viewed 22 positive pictures and rated their pleasure. Each trial began with a 1 s fixation cross, followed by 2 s display positive picture. Participants rated their pleasure within 10 s. After completing all 22 trials, they also took a 5‐min rest.

#### 2.4.2 Testing Phase

There were 20 trials in total, each trial started with a fixation cross for 1 s, participants were sequentially presented with a teachers' common negative emotional scenario, then they were instructed to vividly imagine experiencing each event and regulate their emotions to feel better. After emotion regulation, they rated their pleasure. Following this, participants revisited two personal negative events they previously provided, regulated their emotions, and rated their pleasure using the same scale.

**FIGURE 2:**
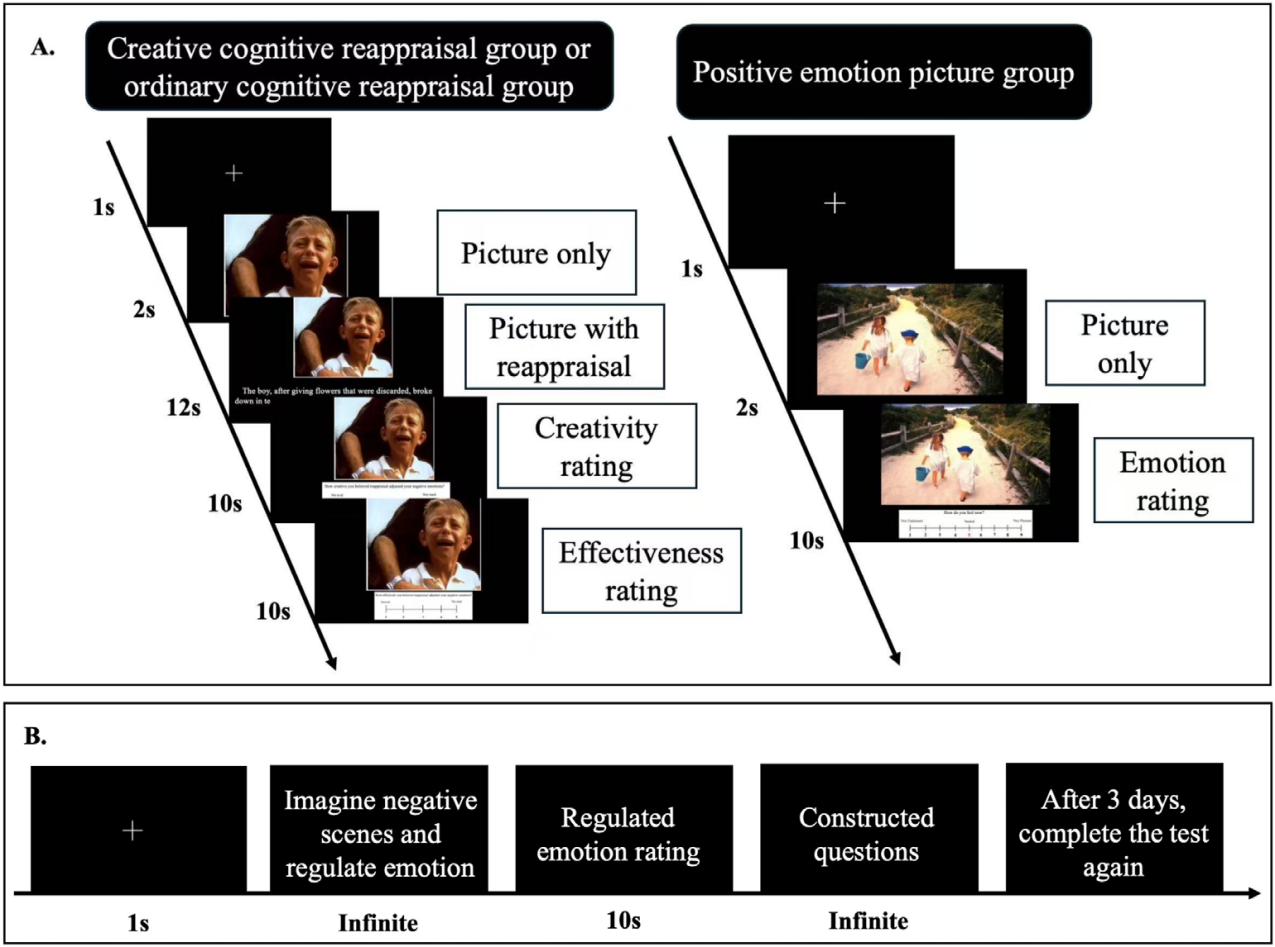
Procedure of experiment. (A) *Learning phase*. (B) *Testing phase*. At the end, they completed a structured questionnaire. The tests of common and personal negative scenes were conducted 3 days later.

After the testing phase, participants completed an open‐ended questionnaire: to assess their emotion regulation strategies, the influence of the learning phase on managing negative emotions, and any insights gained from the learning phase. To examine the long‐term effects of CCR, 3 days later, participants rated their emotional response after regulating the 20 common scenarios and two personal events again (see Figure 2).

### 2.5 Statistical Analyses

Statistical analyses were performed using SPSS 28. A repeated measures ANOVA was conducted to examine group differences across two time points (immediately and 3 days later). Post hoc analyses were conducted to identify significant differences between intervention groups. Additionally, a one‐way ANOVA was used to assess baseline mood differences across the three groups, and Chi‐square tests were employed to evaluate baseline demographic characteristics. If a variable demonstrates a significant difference between groups at baseline, it would be included as a covariate in the analysis.

For the structured questionnaire, two independent raters reviewed participants' answers. The coding criteria are as follows: (1) Emotion regulation strategies (question a): frequency of creative reappraisals. (2) Influence of the learning phase (question b): frequency of the affirmative answer. (3) Insights gained (question c): specific insights reported by participants. The inter‐rater reliability was assessed using Cohen's kappa (Landis and Koch 1977) to ensure consistency. Disagreements were resolved by the supervisor, who re‐evaluated the responses to reach a consensus.

## 3 Results

### 3.1 Demographic Characteristics and Baseline Measures

Men and women were equally distributed among groups (*p* = 0.71), as were teaching grade (*p* = 0.64) and teaching experience (*p* = 0.86). For the baseline mood measure, no significant differences were observed between groups (all *p*s > 0.07), except for anxiety (*p* = 0.014) and worry (*p* = 0.006). These variables were included as covariates in subsequent analyses. No group differences were found for the SAS (*p* = 0.36) or SDS (*p* = 0.46), nor were there differences for the two personal negative events (*p* = 0.93). Detailed demographic characteristics and baseline measures are presented in Tables 1 and 2.

**TABLE 1:** Demographic characteristics.

Dependent variables	CCR (*n* = 27)	OCR (*n* = 26)	PEP (*n* = 29)	$\chi^{2}$	df
Gender				0.69	2
Male	6	8	9		
Female	21	18	20		
Teaching grade				2.56	4
Primary	9	11	15		
Middle	15	13	13		
High	3	2	1		
Teaching years				2.55	6
1–10	10	13	11		
11–20	4	4	3		
21–30	12	9	14		
31–40	1	0	1		

Abbreviations: CCR, creative cognitive reappraisal; OCR, ordinary cognitive reappraisal; PEP, positive emotional picture.

**TABLE 2:** Baseline measures (means and standard deviations).

Dependent variables	CCR (*n* = 27)	OCR (*n* = 26)	PEP (*n* = 29)	F(2, 79)
	*M* (SD)	*M* (SD)	*M* (SD)	
Mood				
Joy	3.52 (1.12)	3.27 (1.04)	3.31 (0.81)	0.49
Calm	3.56 (0.93)	3.50 (1.11)	3.41 (0.73)	0.85
Interest	3.41 (1.08)	3.50 (0.81)	3.17 (0.85)	0.40
Anxiety	2.37 (0.84)	2.81 (0.94)	3.14 (1.06)	4.55*
Worry	2.37 (0.84)	2.81 (0.94)	3.14 (0.83)	5.46**
Anger	2.00 (0.78)	2.27 (0.83)	2.52 (0.87)	2.72
Disappointed	1.78 (0.80)	2.23 (1.11)	2.28 (1.0)	2.18
SAS	41.30 (8.71)	41.59 (8.36)	44.04 (6.41)	1.04
SDS	0.45 (0.10)	0.46 (0.10)	0.48 (0.10)	0.79
Personal events	2.74 (1.30)	2.64 (1.36)	2.64 (0.88)	0.07

Abbreviations: CCR, creative cognitive reappraisal; OCR, ordinary cognitive reappraisal; PEP, positive emotional picture.

* *p <* 0.05.

** *p <* 0.01.

### 3.2 Effect on Dependent Measures

Pleasure ratings were collected twice for 20 common negative scenes and two personal negative events: immediately after learning the phrase, and 3 days later. Descriptive statistics for each group are provided in Table 3.

**TABLE 3:** Means and standard deviations of dependent measures.

Dependent measures	CCR (*n* = 27)	OCR (*n* = 26)	PEP (*n* = 29)	*F*(2, 79)
	*M* (SD)	*M* (SD)	*M* (SD)	
Common scenes				
Pleasure_T1	3.98 (1.07)	4.20 (1.22)	4.01 (1.02)	
Pleasure_T2	4.51 (1.10)	3.82 (1.49)	4.15 (1.04)	
Change scores	0.54 (0.22)	−0.38 (0.22)	0.14 (0.21)	4.51*
Personal scenes				
Pleasure_T1	5.17 (1.42)	5.14 (1.81)	4.67 (1.23)	
Pleasure_T2	5.33 (1.12)	4.56 (1.78)	4.79 (1.22)	
Change scores	0.17 (0.26)	−0.58 (0.27)	0.12 (0.25)	2.47

*Note*: T1 = immediately; T2 = three days later; Change scores = T2‐T1.

Abbreviations: CCR, creative cognitive reappraisal; OCR, original cognitive reappraisal; *M*, mean; PEP, positive emotional picture; SD, standard deviation.

* *p <* 0.05.

#### 3.2.1 Common Negative Scenes

**FIGURE 3:**
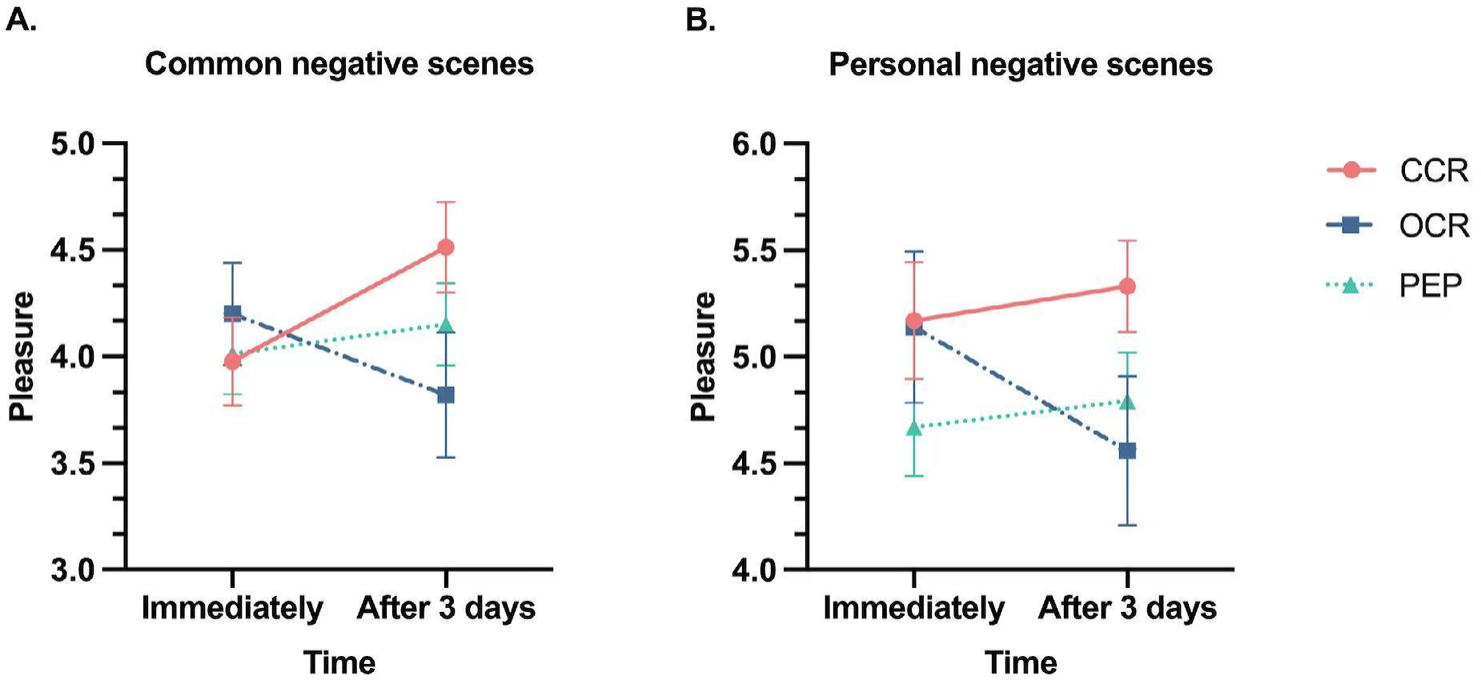
(A) Common negative emotional scenes, and (B) Personal negative emotional scenes. CCR, creative cognitive reappraisal; OCR, ordinary cognitive reappraisal; PEP, positive emotional picture. Error bars represent the stand error.

A significant interaction effect between time and group was observed for the 20 common negative scenes [*F*(2, 79) = 4.51, *p* = 0.014, $\eta_{p}^{2}$ = 0.102] after controlling the baseline anxiety and worry scores. Simple effects analysis indicated that the effect of time was significant for the CCR group [*F*(1, 79) = 6.26, *p* = 0.014, $\eta_{p}^{2}$ = 0.073], with pleasure rating increasing from immediately after the learning phase (*M* = 3.98, SD = 1.07) to 3 days later (*M* = 4.51, SD = 1.10). In contrast, the effect of time was not significant for the OCR group [*F*(1, 79) = 3.03, *p* = 0.09, $\eta_{p}^{2}$ = 0.037], or the positive emotional picture (PEP) group [*F*(1, 79) = 0.46, *p* = 0.50, $\eta_{p}^{2}$ = 0.006] (Figure 3A). Neither the main effect of time [*F*(1, 79) = 0.64, *p* = 0.43, $\eta_{p}^{2}$ = 0.008], nor the main effect of group [*F*(2, 79) = 0.37, *p* = 0.69, $\eta_{p}^{2}$ = 0.009] was significant (see Figure 3A).

#### 3.2.2 Personal Negative Scenes

For the two personal negative events, there was no significant interaction effect between time and group [*F*(2, 79) = 2.47, *p* = 0.09, $\eta_{p}^{2}$ = 0.059] after controlling the baseline anxiety and worry scores. Additionally, neither the main effect of time [*F*(1, 79) = 0.41, *p* = 0.53, $\eta_{p}^{2}$ = 0.005], nor the main effect of group was significant [*F*(2, 79) = 1.25, *p* = 0.29, $\eta_{p}^{2}$ = 0.031]. Specifically, in the CCR group, pleasure rating increased from immediately (*M* = 5.17, SD = 1.42) to 3 days later (*M* = 5.33, SD = 1.12). While, in the OCR group, pleasure rating decreased from immediately (*M* = 5.14, SD = 1.81) to 3 days later (*M* = 4.56, SD = 1.78). In the PEP group, rating showed a slight increase from immediately (*M* = 4.67, SD = 1.23) to 3 days later (*M* = 4.79, SD = 1.22) (see Figure 3B).

### 3.3 Structured Questionnaire

Inter‐rater reliability (kappa values) was good and indicated strong agreement among raters: for emotion regulation strategies (Question a): *k* = 0.85, *p* < 0.001. There were 12 interpretations coded as creative reappraisals in the CCR, while five were in the OCR, and three were in the positive emotional picture (PEP) group.

For influence of the learning phase (Question b): *k* = 0.87, *p* < 0.001. In the CCR, 19 (69%) participants reported being inspired by the learning phase, compared to 15 (58%) in the OCR and 18 (65%) in the PEP group.

For insights gained (Question c): in the CCR group, common themes included “a change of perspective can be helpful in facing the negative events,” “*interesting things can be found even in ugly things or bad situations*,” “things are not as bad as we think.” In the OCR group, participants frequently mentioned “we are so lucky compared to the people in the picture,” “we should be grateful and enjoy the present.” In the PEP group, participants emphasized “*remember to think about the good things in life when feeling down*.” These insights reflect the distinct emotional and cognitive takeaways from each intervention group.

## 4 Discussion

The current study examined the effectiveness of CCR in regulating real‐life negative emotional experiences, addressing the challenges of generating creative reappraisals independently and the limitations of passive comprehension (Wu et al. 2017, 2019). By comparing CCR with OCR and PEP, we assessed pleasure ratings at two time points (immediately, 3 days later) and collected qualitative data to complement the quantitative findings.

This study revealed three key findings. First, for the common negative scenes, the CCR group demonstrated a significant increase in pleasure over time, though no immediate effect was observed, highlighting its potential for transferability and sustained efficacy. This delayed effect suggests that individuals may need time to process and apply cognitive strategies, particularly in emotionally charged states where cognitive resources are limited (Muraven and Baumeister 2000; Sheppes and Meiran 2008). In contrast, the OCR group showed a sharp decline, while the PEP group exhibited only a slight increase. These results confirm that CCR has long‐term benefits (Mac Namara et al. 2011), indicating that repeated use strengthens cognitive restructuring and enhances emotional regulation. However, repeated use of ordinary reappraisal would reduce its effectiveness over time (Meng 2018).

Second, for the personal negative events, the trend was similar to those for common scenes, though no significant differences were observed. Both the CCR and PEP groups showed an increase in pleasure 3 days later, while the OCR group did not. This may be due to the intensity of stimuli differences. Some self‐related events may be so negative that they need more cognitive resources to process (Sheppes and Meiran 2008). This aligns with the idea that the prefrontal cortex, which is critical for cognitive reappraisal, may become dysregulated during intense emotional experiences. Allowing a period of emotional processing can facilitate better regulation later (Sheppes et al. 2011).

Third, for the qualitative results, the CCR group generated creative reappraisals more than twice as high as the OCR and PEP groups (12 vs. 5 vs. 3). Also, the CCR group produced more insights (69%) than the OCR (58%) and PEP (65%) groups, further supporting the quantitative findings. These findings underscore the unique benefits of CCR in fostering meaningful cognitive and emotional shifts. Negative thinking is an automatic response, shaped by years of experience, making it challenging to change (Beck et al. 1979). CCR, a core aspect of cognitive restructuring, helps transform mental representations of negative events effectively, as demonstrated by comparing initial and final solutions (Weisberg 1995).

The findings align with the transfer of training theory (Dweck 2006), which posits that skills learned in one context can be applied to new situations. Participants' ability to transfer CCR strategies from picture‐based learning to real‐life contexts emphasizes its flexibility and generalizability. Additionally, Gross's process model of emotion regulation (Gross 1998) provides a framework for understanding CCR's success: by reframing the meaning of emotionally charged stimuli, CCR increases pleasure, as supported by empirical studies (Ochsner and Gross 2005).

The study's use of real‐life negative scenarios enhances its ecological validity, as it reflects the complex emotional challenges individuals face in everyday life. Barnett and Ceci (2002) highlighted the critical role of context in skill acquisition and application, noting that skills learned in diverse and flexible environment are more likely to be transferred to new situations. CCR holds promise as a tool for fostering resilience across diverse populations and contexts. For example, it could be valuable for individuals navigating setbacks, success lies not in avoiding challenges but in effectively managing them, like the proverb, “*Success in life is not about holding good cards, but playing bad cards well*.” This broader applicability positions CCR as a valuable resource for personal growth and emotional resilience in real‐world settings.

## 5 Conclusion and Future Studies

Our findings indicate that CCR exhibits a meaningful and delayed effect supported quantitatively and qualitatively after 3 days. This suggests that CCR is an effective, teachable, and enduring skill, offering a long‐term benefit for regulating negative emotion in real‐world contexts. The delayed effect may reflect the importance of allowing time for emotional processing—rather than attempting immediate regulation—which can create a pathway for more effective regulation later. This underscores the crucial role of timing and emotional awareness in emotion regulation strategies. By first acknowledging and processing intense emotions, individuals may be better equipped to engage in cognitive reappraisal successfully, ultimately leading to healthier emotional outcomes. These insights advance theories on creative reappraisal and have practical implications for psychological counseling and emotional education programs.

Several areas for improvement should be addressed. First, future research should explore ways to maximize its immediate and long‐term effect. Second, teaching experience and age are likely critical determinants of emotion regulation capacities among teachers. Further research should consider these variables in the analysis. Third, to better understand the durability and trajectory of the intervention's effects, future studies could adopt longitudinal design to examine the longer follow‐up intervals (e.g., 7, 14 days, or 1 month), which would provide more comprehensive evidence regarding the practical applications in educational or therapeutic settings. Additionally, examining individual differences, such as personality traits or baseline emotional regulation skills, may help identify who benefits most from this intervention and optimize a person‐situation fit strategy for regulating negative emotions.

## Disclosure

The content has not been published or submitted for publication elsewhere. All authors have contributed significantly, and all authors agree with the content of the manuscript.

## Conflicts of Interest

The authors declare no conflicts of interest.

## Data Availability

The data supporting the findings of this study will be made available, upon reasonable request.
